# A Social Identity Approach to Understanding and Promoting Physical Activity

**DOI:** 10.1007/s40279-017-0720-4

**Published:** 2017-03-27

**Authors:** Mark Stevens, Tim Rees, Pete Coffee, Niklas K. Steffens, S. Alexander Haslam, Remco Polman

**Affiliations:** 1 Department of Sport and Physical Activity, Faculty of Management, Bournemouth University, Dorset House, Talbot Campus, Fern Barrow, Poole, BH12 5BB UK; 2 Faculty of Health Sciences and Sport, University of Stirling, Stirling, UK; 3 School of Psychology, The University of Queensland, McElwain Building, St Lucia, QLD 4072 Australia; 4 Department of Psychology, Faculty of Science and Technology, Bournemouth University, Dorset House, Talbot Campus, Fern Barrow, Poole, BH12 5BB UK

## Abstract

Against the backdrop of a global physical inactivity crisis, attempts to both understand and positively influence physical activity behaviours are characterized by a focus on individual-level factors (e.g. cognitions, attitudes, motivation). We outline a new perspective, drawn from an emerging body of work exploring the applicability of social identity and self-categorization theories to domains of sport and health, from which to understand and address this pervasive problem. This social identity approach suggests that the groups to which people belong can be, and often are, incorporated into their sense of self and, through this, are powerful determinants of physical activity-related behaviour. We start by reviewing the current state of physical activity research and highlighting the potential for the social identity approach to help understand how social factors influence these behaviours. Next, we outline the theoretical underpinnings of the social identity approach and provide three key examples that speak to the analytical and practical value of the social identity approach in physical activity settings. Specifically, we argue that social identity (1) can be harnessed to promote engagement in physical activity, (2) underpins exercise group behaviour, and (3) underpins effective leadership in exercise settings. We conclude by identifying prospects for a range of theory-informed research developments.

## Key Points

**Table:** 

Social factors have a significant impact on physical activity behaviours, and our understanding of their influence will be improved by applying theories of group behaviour to this context.
The social identity approach provides a valuable framework from which to explore the impact of social factors on physical activity behaviours.
Through three broad examples, we illustrate how the social identity approach has the potential to enrich both theory and practice in the physical activity domain.

## Introduction

In this article, we highlight the potential for a social identity approach to advance understanding and promotion of physical activity behaviours.^1^ Since the 1970s, this approach has been applied to a vast array of contexts, including politics [2–5], business and organizations [6], sport [7], and, of particular relevance to the present article, health [8–11]. One of the key propositions of the social identity approach is that psychology and behaviour are both heavily structured by the group memberships that individuals internalise as part of their sense of self [12, 13]. Indeed, because physical activity is often conducted within group settings (e.g. Nordic walking groups, exercise groups, and sports teams), it represents a domain to which a social identity approach could have particular relevance.

In the sections that follow, we first explore current approaches to understanding and promoting physical activity. Despite their differences, these converge in highlighting a need for greater consideration of the impact of social factors on health-related behaviours. We then provide a brief introduction to the social identity approach, before offering three examples of how the approach can fruitfully contribute to understanding and application in the field of physical activity.

## Physical Activity, Health, and Participation Rates

The influence of physical activity on health and well-being is well documented. Physiological benefits include a reduced risk of contracting coronary heart disease [14, 15], developing various types of cancers [16], and having a stroke [17]; psychological benefits include reduced anxiety [18, 19], reduced likelihood of depression [20, 21], and improved self-esteem [22]. Conversely, physical inactivity has been identified as the fourth leading cause of death worldwide [23], with estimates suggesting that, of all deaths from non-communicable diseases, 6–10% can be attributed to physical inactivity [24]. Enhancing long-term participation in physical activity has consequently been identified as a key objective for researchers, government-funded organizations, and public health agencies. For example, World Health Organization member states have agreed a plan to target a 10% reduction in physical inactivity by 2025 [25]. However, notwithstanding attempts to address this problem, participation rates remain poor; global data from 146 countries suggest that almost one-quarter of adults (23.3%) worldwide are insufficiently active [26].

## Current Approaches to Understanding and Promoting Physical Activity

Given this physical inactivity pandemic [23, 26], considerable effort has been devoted to understanding physical activity behaviours. Indeed, research concerning the correlates and determinants of physical activity has accelerated in the past 2 decades. Concentrating largely on demographic or individual factors such as age, sex, health status, cognitions, attitudes, and motivation [27], this research has often explored the capacity for theories that predominantly focus on individuals as individuals, such as self-determination theory [28] and the theory of planned behaviour [29], to predict and explain behaviour change [30–32].^2^

Similarly, interventions to promote physical activity have generally employed individual-level psychological and cognitive–behavioural strategies, such as education, self-monitoring, cognitive restructuring, and goal setting [35–37]. Such efforts often also involve attempting to progress individuals through specified stages of behaviour change—for example, as described in the transtheoretical model (TTM) [38]. Although there is some evidence for both the efficacy of these techniques [39] and the predictive utility of these models [40], support for the TTM is relatively weak [41], with mixed findings emerging from studies examining its utility as a predictor of behaviour change and as a basis for intervention [42, 43].

Trends over time indicate that physical activity levels remain stagnant at best and may even be decreasing. Indeed, in the USA, for example, physical *in*activity rates among people aged ≥6 years increased by 0.9% between 2010 and 2015 [44]. The latest data also suggest that worldwide physical activity levels are not increasing, despite many countries having a national physical activity policy or plan [28]. Furthermore, meta-analyses of physical activity interventions have often reported small overall effect sizes [45, 46] and large heterogeneity in effect size strength [47]. All these trends suggest that, despite the considerable volume of research that has been conducted, further work is still required to identify—and mobilize—the most effective strategies for behaviour change in this domain.

## Recent Advances in Understanding Behaviour Change

Researchers have recently explored new avenues in attempting to advance understanding of behaviour change, including the development of taxonomies of the numerous strategies that have been employed in the context of smoking cessation [48], alcohol consumption [49], and healthy eating and physical activity [50, 51]. Researchers have also explored (1) how best to frame behaviour change messages [52, 53], (2) the utility of new mobile and sensing technologies [54, 55], and (3) the relationship between affective responses to exercise and exercise adherence [56].

In addition to these new lines of enquiry, researchers have begun to acknowledge the importance of moving beyond an exclusive focus on individual-level approaches to behaviour change to ecological models that consider the numerous individual, environmental, policy, and social determinants of health behaviours [27, 33, 57]. Representing an important shift from traditional theoretical approaches, the assumption at the heart of these models is that understanding behaviour change at different levels (e.g. both individual and collective) is critical for the development of successful interventions [57]. By way of example, initial findings identify the physical activity benefits associated with attending to, and engaging with, a person’s social support and social capital, and the norms that develop in group contexts [58, 59]. Research has also shown that when people possess more favourable perceptions regarding ‘protective social factors’ in their communities (e.g. in relation to the quality of social networks, the degree of social cohesion, and the level of trust in neighbours) they are more likely to engage in physical activity [60, 61].

Similar findings have been documented in the broader health domain, with research consistently demonstrating the impact of social factors on individuals’ mental and physical health [62, 63]. Of particular relevance to the present article, research informed by the social identity approach has also emphasized the health benefits (both mental and physical) that accrue from people possessing, maintaining, and developing social identities derived from meaningful memberships of social groups [10]. In particular, research has shown that internalized social group memberships have positive effects on health in a range of contexts—including choirs [64], care homes [65], and, of most interest to this article, sports teams [66]. In these various settings, increased social identification has also been shown to have positive consequences for mental health-based indicators of self-esteem [67], quality of life [68], depression [69], and stress [70]. To flesh these ideas out, in the sections that follow, we provide a brief introduction to the social identity approach,^3^ followed by three illustrative applications of the approach to the field of physical activity.

## The Social Identity Approach

The social identity approach comprises two theories: social identity theory [12, 71, 72] and self-categorization theory [73–76]. The broad goal of the approach is to provide a comprehensive analysis of the way in which individual psychology is structured by group life. The approach starts by recognizing that individuals can define themselves, and behave, not only as individuals (in terms of personal identity as ‘I’ and ‘me’ [73]) but also as group members (in terms of social identity as ‘we’ and ‘us’). Moreover, it proposes that when people categorize themselves as members of a group, this gives their behaviour a distinct meaning, in part because it motivates them to positively differentiate their ingroup from comparison outgroups on valued dimensions. That is, when an individual’s sense of who they are is defined in terms of ‘we’ rather than ‘I’, they strive to see ‘us’ as special and as different from other groups [6].

According to this approach, group behaviour is associated with a change in the structure of the self whereby, through a process of depersonalization, the self comes to be perceived as categorically interchangeable with other ingroup members [74]. Defining oneself in terms of a specific social identity is associated with a desire both to discover the meaning of that identity and to align one’s attitudes and behaviours with others who share it [6, 76]. So, for example, the more a person identifies with a gym class or exercise group (e.g. as a CrossFit exerciser), a running group (e.g. as a parkrunner), or a team (e.g. as a soccer player of team X), the more that person will be motivated to discover and align themselves with the norms, values, and ideals of what it means to be a member of that group.

## The Social Identity Approach Applied to Physical Activity

### Social Identity can be Harnessed to Promote Engagement in Physical Activity

In line with the foregoing arguments, research by Terry and Hogg [77] found that individuals who identified strongly with a group in which exercise was normative reported greater intentions to engage in regular exercise than those who identified weakly with the group. These findings have subsequently been supported by a large body of experimental research in the broader health domain, which has shown that people are more likely to engage in healthy behaviours if, and to the extent that, these are congruent with the content of a salient social identity [78, 79]. For example, young adults report weaker intentions to reduce alcohol consumption when their social identity as a ‘university student’ rather than as a ‘British person’ is made salient [79]. Showing too that identity-based intentions translate into identity-congruent behaviour, Strachan et al. [80] found that runners who identified more strongly with their running group completed a greater proportion of their runs with the group but were less confident they would continue running should the group disband. Complementing both self-determination theory [28] and the theory of planned behaviour [29], these findings reinforce the notion that intentions predict behaviour. Crucially, however, they extend this proposition by demonstrating that this effect is particularly strong when those intentions are structured by internalized social identities.

Other research informed by the social identity approach has extended these ideas by highlighting the importance of the structure of exercise environments in fostering identity development. Across multiple studies, Beauchamp and colleagues [81, 82] and Dunlop and Beauchamp [83, 84] have shown that people feel more inclined to exercise with others with whom they share membership in a particular social category (e.g. as ‘us women’). Among other things, these researchers found that age and sex are particularly common markers of shared social identity in exercise settings and that participants who perceived themselves to be similar to other group members in terms of physical characteristics (i.e. age, physical appearance, and physical condition) displayed greater levels of adherence to an exercise programme than those who perceived themselves to be dissimilar to other group members [83].

Such findings suggest that people seek out and create ingroups (and outgroups) in exercise settings [85] and that the opportunity to exercise with other ingroup (rather than outgroup) members is therefore an important determinant of their continued engagement in exercise [86]. They also suggest that people who design exercise programmes need to attend to both (1) the opportunities these provide for emergent social identities and (2) the ways in which the programme allows these identities to be enacted and maintained (e.g. through interaction with ingroup members).

Supporting these assertions, a recent randomized controlled trial of the Football Fans in Training (FFIT) programme revealed a significant 4.36% difference in percentage weight loss between intervention and control groups at 12-month follow-up [87]. FFIT is a 12-week programme delivered exclusively to overweight male football fans to improve their diet and physical activity. Crucially, participants share a common social identity as fans of the same team, with interaction between ingroup members assured. Such interaction is also facilitated within many other recently developed exercise programmes (e.g. ‘Baby Bootcamp’, ‘Karate 4 Kids’, ‘Swimming for Seniors’), suggesting the value of social identities is already well understood (albeit implicitly) by their initiators.

These various lines of research all speak to the idea that social identities can have profound implications for participation in, and adherence to, physical activity. However, as yet, the body of research that supports such claims is relatively small. Moreover, it is further limited by a predominant focus on healthy, non-clinical populations. Given the additional barriers to participation experienced by clinical populations (e.g. lack of mobility, reliance on carers), research examining the impact of social identity within clinical exercise settings (e.g. cardiac rehabilitation, obesity care, disability groups) would represent a valuable adjunct to continued non-clinical research. Indeed, such groups would represent a unique challenge to programmes designed to provide opportunities for social identities to emerge and be harnessed.

### Social Identity Underpins Exercise Group Behaviour

Examination of the benefits of group exercise environments, where multiple individuals undertake the same structured exercise activity, is not new. Indeed, the effectiveness of interventions that involve individual- and group-based exercise environments have been studied extensively, with good evidence that group environments are more effective than individual environments in promoting adherence. Efforts to develop cohesiveness within exercise groups have proved particularly effective [88]. Research across multiple settings and populations has demonstrated a range of positive outcomes from exercising in so-called ‘true groups’ where group dynamics principles have been used to increase cohesiveness [88]. Most notably, these benefits include long-term increases in physical activity [89–91] (see Estabrooks et al. [92]; Harden et al. [93] for recent reviews).

Research examining the effectiveness of these ‘true groups’ also reveals that successful interventions foster the development of social identity. For example, the influential model by Carron and Spink [94] proposes that a sense of distinctiveness plays an important role in motivating members of exercise groups to engage in group-relevant activity (see also Bruner and Spink [95, 96]). Clarifying the causal role of social identification in these outcomes, experimental research that enhanced social identification by providing group t-shirts and encouraging participants to develop a group name found this led to greater subsequent effort in a group task [97].

Such findings suggest that social identity is a key mechanism that underpins the effectiveness of group-based programmes in exercise settings. Again, though, this hypothesis is yet to be extensively tested. In particular, there is a need for much more empirical research to explore the role that social identities play in the effectiveness of various forms of exercise groups, interventions, and programmes in the world at large (e.g. gym membership, CrossFit, parkrun).

### Social Identity Underpins Effective Leadership in Exercise Settings

According to the social identity approach, it is the shift in self-categorization from a personal to a social identity that underpins social collaboration and indeed all forms of group behaviour [73]. Extending this reasoning, social identity theorizing contends that, when people categorize themselves as members of the same group (i.e. in terms of shared social identity), this provides the basis for mutual social influence [75]. However, at the same time, the capacity for any given individual to exert influence varies as a function of his or her capacity to represent and embody the meaning of the group in a given social context. Put slightly differently, this means that any individual group member’s ability to exert leadership depends on his or her ingroup prototypicality [98–100].

More generally, from a social identity perspective, successful leadership depends on a leader’s ability to create, represent, advance, and embed a shared sense of identity among group members [99, 101]. In line with this idea, evidence suggests that exercise leaders are more likely to have a positive role in shaping the affective states and effectiveness of group members’ behaviours if they both stand for, and stand up for, the group [102, 103].

Although the efficacy of the social identity approach to leadership has yet to be extensively examined in exercise settings, a vast body of other research supports its applicability to this context. Benefits associated with identity leadership in other (mainly organizational) contexts include increased satisfaction [104–106], effort [107, 108], and support for leaders [98, 109, 110] as well as reduced turnover intentions [105, 106] and burnout [111]. Such findings appear to have clear relevance to exercise settings. For example, higher levels of burnout have been extensively linked to motivation loss and dropout among sports team players [112–115], emphasizing the value of minimizing the occurrence of burnout in exercise settings.

Finally, the social identity perspective suggests that, before an individual can lead a group, he/she first has to understand it [100]. This suggests there would be particular value in exercise leaders (1) taking opportunities to learn about group history, culture, and functioning and (2) attending to collective group values, norms, and goals. Understanding these nuanced dimensions of group identity will enhance their capacity to be perceived as a prototypical group member and thus engender support (e.g. through demonstrating a level of effort congruent with the expectations and desires of group members) and facilitate the achievement of group and individual goals (e.g. through devising and delivering appropriate group sessions).

Again, though, empirical tests of the identity leadership approach in clinical and non-clinical exercise settings are now needed to confirm its seemingly substantial potential and to identify factors that moderate (i.e. either facilitate or stifle) its impact. Aspects of the approach may, for example, be less applicable in clinical settings (e.g. cardiac rehabilitation), where medical expertise may be favoured over leader prototypicality. However, at the same time, the relative value of leaders helping to create an appropriate identity for such a group (e.g. in which supportiveness and celebrating others’ progress is considered normative) may be substantial. These nuances await research. Indeed, the research Steffens et al. [111] conducted in an organizational setting represented the first attempt to explore the role of social identity as a lynchpin between leadership and health. Nevertheless, Wegge et al. [116] suggested this might “have merely exposed the tip of what is a large theoretical iceberg.” Building on these sentiments, we believe the approach has an equally significant potential in exercise contexts where health and well-being are even more centre stage.

## Conclusion

The social identity approach represents a potentially fruitful but greatly under-examined framework for understanding and promoting physical activity. It also presents a viable alternative to the individualistic treatments that currently dominate the theoretical landscape. In the limited space available here, we have provided three brief illustrations of the ways in which this approach might enrich theory and practice. Our hope is that, though barely sketched out here, the framework we have outlined will serve as the foundation for an exciting new wave of original research into the role that group and identity dynamics play in shaping physical activity behaviours. Certainly, the clear applicability of the approach to this domain, and the substantial contribution it has already made in others, makes us confident that the approach has the capacity to drive a groundswell of empirical research, and that the advances this would yield would be considerable.

## Footnotes

1 We consider physical activity in the widest sense, including exercise and sport participation. We use the term ‘exercise’ where applicable throughout the article when referring specifically to physical activity that is planned, structured, and repetitive, with the aim of maintaining or improving physical fitness [1].

2 Although we note that these theories do mention social factors (e.g. related to the notions of subjective norms, integrated regulation), they lack an analysis of the self as derived from social groups in a social context [33, 34].

3 This brief introduction to the social identity approach should not be considered a treatise on the topic; readers are referred to Haslam [6] and Rees et al. [7] for detailed explications of the approach.

## References

[1] Caspersen CJ, Powell KE, Christenson GM (1985). Physical activity, exercise, and physical fitness: definitions and distinctions for health-related research. Public Health Rep.

[2] Augoustinos M, De Garis S (2012). ‘Too black or not black enough’: social identity complexity in the political rhetoric of Barack Obama. Eur J Soc Psychol..

[3] Reicher S, Hopkins N (1996). Self-category constructions in political rhetoric; an analysis of Thatcher’s and Kinnock’s speeches concerning the British miners’ strike (1984-5). Eur J Soc Psychol..

[4] Reicher S, Hopkins N (2000). Self and nation.

[5] Steffens NK, Haslam SA (2013). Power through ‘us’: leaders’ use of we-referencing language predicts election victory. PLoS One.

[6] Haslam SA (2004). Psychology in organizations: the social identity approach.

[7] Rees T, Haslam SA, Coffee P (2015). A social identity approach to sport psychology: principles, practice, and prospects. Sports Med.

[8] Greenaway KH, Cruwys T, Haslam SA (2016). Social identities promote well-being because they satisfy global psychological needs. Eur J Soc Psychol..

[9] Haslam C, Cruwys T, Haslam SA (2016). Groups 4 Health: evidence that a social-identity intervention that builds and strengthens social group membership improves mental health. J Affect Disorders..

[10] Jetten J, Haslam C, Haslam SA (2012). The social cure: identity, health and well-being.

[11] Steffens NK, Haslam SA, Schuh SC (2016). A meta-analytic review of social identification and health in organizational contexts. Pers Soc Psychol Rev..

[12] Tajfel H, Turner JC, Austin WG, Worchel S (1979). An integrative theory of intergroup conflict. The social psychology of intergroup relations.

[13] Turner JC, Oakes PJ, Haslam SA (1994). Self and collective: cognition and social context. Pers Soc Psychol Bull.

[14] Berlin JA, Colditz GA (1990). A meta-analysis of physical activity in the prevention of coronary heart disease. Am J Epidemiol.

[15] Sattelmair J, Pertman J, Ding EL (2011). Dose response between physical activity and risk of coronary heart disease: a meta-analysis. Circulation.

[16] Lee IM (2003). Physical activity and cancer prevention—data from epidemiologic studies. Med Sci Sport Exerc.

[17] Do Lee C, Folsom AR, Blair SN (2003). Physical activity and stroke risk: a meta-analysis. Stroke.

[18] Petruzzello SJ, Landers DM, Hatfield BD (1991). A meta-analysis on the anxiety-reducing effects of acute and chronic exercise. Outcomes and mechanisms. Sports Med..

[19] Taylor AH, Biddle SJH, Fox KR, Boutcher SH (2003). Physical activity, anxiety, and stress. Physical activity and psychological well-being.

[20] Dunn AL, Trivedi MH, O’Neal HA (2001). Physical activity dose-response effects on outcomes of depression and anxiety. Med Sci Sport Exerc..

[21] Teychenne M, Ball K, Salmon J (2008). Physical activity and likelihood of depression in adults: a review. Prev Med.

[22] Ekeland E, Heian F, Hagen KB (2005). Can exercise improve self esteem in children and young people? A systematic review of randomised controlled trials. Br J Sports Med.

[23] Kohl HW, Craig CL, Lambert EV (2012). The pandemic of physical inactivity: global action for public health. Lancet.

[24] Lee IM, Shiroma EJ, Lobelo F (2012). Effect of physical inactivity on major non-communicable diseases worldwide: an analysis of burden of disease and life expectancy. Lancet.

[25] World Health Organization. Physical inactivity: Meeting the 2025 global targets. Geneva: WHO; 2013. http://www.searo.who.int/entity/noncommunicable_diseases/events/ncd_workshop_2014_physical_inactivity.pdf. Accessed 19 Nov 2016.

[26] Sallis JF, Bull F, Guthold R (2016). Progress in physical activity over the Olympic quadrennium. Lancet.

[27] Bauman AE, Reis RS, Sallis JF (2012). Correlates of physical activity: why are some people physically active and others not?. Lancet.

[28] Deci EL, Ryan RM (1985). Intrinsic motivation and self-determination in human behavior.

[29] Ajzen I (1991). The theory of planned behavior. Organ Behav Hum Dec..

[30] Armitage CJ (2005). Can the theory of planned behavior predict the maintenance of physical activity?. Health Psychol.

[31] Hagger MS, Chatzisarantis NL, Biddle SJH (2002). A meta-analytic review of the theories of reasoned action and planned behavior in physical activity: predictive validity and the contribution of additional variables. J Sport Exerc Psychol..

[32] Teixeira PJ, Carraça EV, Markland D (2012). Exercise, physical activity, and self-determination theory: a systematic review. Int J Behav Nutr Phys Act..

[33] Cruwys T, Haslam SA, Fox N (2015). “That’s not what we do”: evidence that normative change is a mechanism of action in group interventions. Behav Res Ther.

[34] Terry DJ, Hogg MA, White KM (1999). The theory of planned behaviour: self identity, social identity and group norms. Br J Soc Psychol.

[35] Greaves CJ, Sheppard KE, Abraham C (2011). Systematic review of reviews of intervention components associated with increased effectiveness in dietary and physical activity interventions. BMC Public Health..

[36] Speake H, Copeland RJ, Till SH (2016). Embedding physical activity in the heart of the NHS: the need for a whole-system approach. Sports Med.

[37] Tully MA, Hunter RF (2015). Promoting physical activity: time for a major re-think. Aspetar Sports Med J..

[38] Prochaska JO, DiClemente CC (1983). Stages and processes of self-change of smoking: toward an integrative model of change. J Consult Clin Psychol.

[39] Shilts MK, Horowitz M, Townsend MS (2004). Goal setting as a strategy for dietary and physical activity behavior change: a review of the literature. Am J Health Promot..

[40] Marshall SJ, Biddle SJ (2001). The transtheoretical model of behavior change: a meta-analysis of applications to physical activity and exercise. Ann Behav Med.

[41] National Institute for Health and Care Excellence. Behaviour change: Individual approaches. London: NICE; 2014. https://www.nice.org.uk/guidance/ph49/resources/behaviour-change-individual-approaches-1996366337989. Accessed 19 Nov 2016.

[42] Adams J, White M (2005). Why don’t stage-based activity promotion interventions work?. Health Educ Res.

[43] Bridle C, Riemsma RP, Sowden AJ (2005). Systematic review of the effectiveness of health behavior interventions based on the transtheoretical model. Psychol Health..

[44] Physical Activity Research Council. The Physical Activity Council’s annual study tracking sports, fitness and recreation participation in the US. Physical Activity Research Council; 2016. http://www.physicalactivitycouncil.com/pdfs/current.pdf. Accessed 19 Nov 2016.

[45] Conn VS, Valentine JC, Cooper HM (2002). Interventions to increase physical activity among aging adults: a meta-analysis. Ann Behav Med.

[46] Harris KC, Kuramoto LK, Schulzer M (2009). Effect of school-based physical activity interventions on body mass index in children: a meta-analysis. CMAJ.

[47] Conn VS, Hafdahl AR, Cooper PS (2009). Meta-analysis of workplace physical activity interventions. Am J Prev Med.

[48] Michie S, Hyder N, Walia A (2011). Development of a taxonomy of behaviour change techniques used in individual behavioural support for smoking cessation. Addict Behav.

[49] Michie S, Whittington C, Hamoudi Z (2012). Identification of behaviour change techniques to reduce excessive alcohol consumption. Addiction.

[50] Abraham C, Michie S (2008). A taxonomy of behavior change techniques used in interventions. Health Psychol.

[51] Michie S, Ashford S, Sniehotta FF (2011). A refined taxonomy of behaviour change techniques to help people change their physical activity and healthy eating behaviours: the CALO-RE taxonomy. Psychol Health..

[52] Gerend MA, Maner JK (2011). Fear, Anger, fruits, and veggies: interactive effects of emotion and message framing on health behavior. Health Psychol.

[53] Gerend MA, Shepherd MA (2016). When different message frames motivate different routes to the same health outcome. Ann Behav Med.

[54] King AC, Ahn DK, Oliveira BM (2008). Promoting physical activity through hand-held computer technology. Am J Prev Med.

[55] King AC, Hekler EB, Grieco LA (2013). Harnessing different motivational frames via mobile phones to promote daily physical activity and reduce sedentary behavior in aging adults. PLoS One.

[56] Ekkekakis P, Parfitt G, Petruzzello SI (2011). The pleasure and displeasure people feel when they exercise at different intensities: decennial update and progress towards a tripartite rationale for exercise intensity prescription. Sports Med.

[57] Sallis JF, Owen N, Fisher EB, Glanz K, Rimer BK, Viswanath K (2008). Ecological models of health behavior. Health behavior and health education: theory, research, and practice.

[58] Broyles ST, Mowen AJ, Theall KP (2011). Integrating social capital into a park-use and active-living framework. Am J Prev Med.

[59] Resnick B, Orwig D, Magaziner J (2002). The effect of social support on exercise behavior in older adults. Clin Nurs Res..

[60] Brennan LK, Baker EA, Haire-Joshu D (2003). Linking perceptions of the community to behavior: are protective social factors associated with physical activity?. Health Educ Behav..

[61] Kaczynski AT, Glover TD (2012). Talking the talk, walking the walk: examining the effect of neighbourhood walkability and social connectedness on physical activity. J Public Health..

[62] Ho CY (2016). Better health with more friends: the role of social capital in producing health. Health Econ.

[63] Wong ST, Wu A, Gregorich S (2014). What type of social support influences self-reported physical and mental health among older women?. J Aging Health.

[64] Dingle GA, Brander C, Ballantyne J (2013). ‘To be heard’: the social and mental health benefits of choir singing for disadvantaged adults. Psychol Music.

[65] Haslam C, Haslam SA, Knight C (2014). We can work it out: group decision-making builds social identity and enhances the cognitive performance of care residents. Br J Psychol.

[66] Jones JM, Jetten J (2011). Recovering from strain and enduring pain: multiple group memberships promote resilience in the face of physical challenges. Soc Psychol Pers Sci..

[67] Jetten J, Branscombe NR, Haslam SA (2015). Having a lot of a good thing: multiple important group memberships as a source of self-esteem. PLoS One.

[68] Steffens NK, Cruwys T, Haslam C (2016). Social group memberships in retirement are associated with reduced risk of premature death: evidence from a longitudinal cohort study. BMJ Open.

[69] Cruwys T, Haslam SA, Dingle GA (2014). Depression and social identity: an integrative review. Pers Soc Psychol Rev..

[70] Haslam SA, Reicher S (2006). Stressing the group: social identity and the unfolding dynamics of responses to stress. J Appl Psychol.

[71] Tajfel H, Moscovici S (1972). Social categorization. Introduction a la psychologie sociale.

[72] Tajfel H (1974). Social identity and intergroup behaviour. Soc Sci Inf..

[73] Turner JC, Tajfel H (1982). Towards a cognitive redefinition of the social group. Social identity and intergroup relations.

[74] Turner JC, Lawler EJ (1985). Social categorization and the self-concept: A social cognitive theory of group behaviour. Advances in group processes (vol 2).

[75] Turner JC (1991). Social influence.

[76] Turner JC, Hogg MA, Oakes PJ (1987). Rediscovering the social group: a self-categorization theory.

[77] Terry DJ, Hogg MA (1996). Group norms and the attitude-behavior relationship: a role for group identification. Pers Soc Psychol Bull.

[78] Oyserman D, Fryberg SA, Yoder N (2007). Identity-based motivation and health. J Pers Soc Psychol.

[79] Tarrant M, Butler K (2011). Effects of self-categorization on orientation towards health. Br J Soc Psychol.

[80] Strachan SM, Shields CA, Glassford A (2012). Role and group identity and adjustment to the possibility of running group disbandment. Psychol Sport Exerc.

[81] Beauchamp MR, Carron AV, McCutcheon S (2007). Older adults’ preferences for exercising alone versus in groups: considering contextual congruence. Ann Behav Med.

[82] Beauchamp M, Dunlop WL, Downey SM (2012). First impressions count: perceptions of surface-level and deep-level similarity within postnatal exercise classes and implications for program adherence. J Health Psychol..

[83] Dunlop WL, Beauchamp MR (2011). Does similarity make a difference? Predicting cohesion and attendance behaviors within exercise group settings. Group Dyn..

[84] Dunlop WL, Beauchamp MR (2011). En-gendering choice: preferences for exercising in gender-segregated and gender-integrated groups and consideration of overweight status. Int J Behav Med..

[85] Bruner MW, Dunlop WL, Beauchamp MR, Beauchamp MR, Eys MA (2014). A social identity perspective on group processes in sport and exercise. Group dynamics in exercise and sport psychology.

[86] Beauchamp MR, Harden SM, Wolf SA (2015). GrOup based physical Activity for oLder adults (GOAL) randomized controlled trial: study protocol. BMC Public Health.

[87] Hunt K, Wyke S, Gray CM (2014). A gender-sensitised weight loss and healthy living programme for overweight and obese men delivered by Scottish Premier League football clubs (FFIT): a pragmatic randomised controlled trial. Lancet.

[88] Burke SM, Carron AV, Eys MA (2006). Group versus individual approach? A meta-analysis of the effectiveness of interventions to promote physical activity. Sport Exerc Psychol Rev..

[89] Estabrooks PA, Bradshaw M, Dzewaltowski DA (2008). Determining the impact of Walk Kansas: applying a team-building approach to community physical activity promotion. Ann Behav Med.

[90] Estabrooks PA, Almeida FA, Hill J (2011). Move more: translating an efficacious group dynamics physical activity intervention into effective clinical practice. Int J Sport Exerc Psychol..

[91] Rejeski WJ, Brawley LR, Ambrosius WT (2003). Older adults with chronic disease: benefits of group-mediated counseling in the promotion of physically active lifestyles. Health Psychol.

[92] Estabrooks PA, Harden SM, Burke SM (2012). Group dynamics in physical activity promotion: what works?. Soc Pers Psychol Compass.

[93] Harden SM, McEwan D, Sylvester BD (2015). Understanding for whom, under what conditions, and how group-based physical activity interventions are successful: a realist review. BMC Public Health.

[94] Carron AV, Spink KS (1993). Team building in an exercise setting. Sport Psychol..

[95] Bruner MW, Spink KS (2010). Evaluating a team building intervention in a youth exercise setting. Group Dyn..

[96] Bruner MW, Spink KS (2011). Effects of team building on exercise adherence and group task satisfaction in a youth activity setting. Group Dyn..

[97] Høigaard R, Boen F, De Cuyper B (2013). Team identification reduces social loafing and promotes social laboring in cycling. Int J Appl Sports Sci..

[98] Haslam SA, Reicher S (2007). Identity entrepreneurship and the consequences of identity failure: the dynamics of leadership in the BBC prison study. Soc Psychol Q.

[99] Haslam SA, Reicher SD, Platow MJ (2010). The new psychology of leadership: Identity, influence, and power.

[100] Hogg MA (2001). A social identity theory of leadership. Pers Soc Psychol Rev..

[101] Steffens NK, Haslam SA, Jetten J (2014). Leadership as social identity management: introducing the Identity Leadership Inventory (ILI) to assess and validate a four-dimensional model. Leadersh Q.

[102] Fransen K, Haslam SA, Steffens NK (2015). Believing in “us”: exploring leaders’ capacity to enhance team confidence and performance by building a sense of shared social identity. J Exp Psychol Appl..

[103] Fransen K, Steffens NK, Haslam SA (2015). We will be champions: leaders’ confidence in ‘us’ inspires team members’ team confidence and performance. Scand J Med Sci Sports.

[104] Cicero L, Pierro A, van Knippenberg D (2007). Leader group prototypicality and job satisfaction: the moderating role of job stress and team identification. Group Dyn..

[105] Cicero L, Pierro A, van Knippenberg D (2010). Leadership and uncertainty: how role ambiguity affects the relationship between leader group prototypicality and leadership effectiveness. Br J Manage..

[106] Pierro A, Cicero L, Bonaiuto M (2005). Leader group prototypicality and leadership effectiveness: the moderating role of need for cognitive closure. Leadersh Q.

[107] Cicero L, Bonaiuto M, Pierro A (2008). Employees’ work effort as a function of leader group prototypicality: the moderating role of team identification. Eur Rev Appl Psychol..

[108] León JAM, Cantisano GT (2009). Mangin J-PL. Leadership in nonprofit organizations of Nicaragua and El Salvador: a study from the social identity theory. Span J Psychol..

[109] Platow MJ, Hoar S, Reid S (1997). Endorsement of distributively fair and unfair leaders in interpersonal and intergroup situations. Eur J Soc Psychol..

[110] Ullrich J, Christ O, van Dick R (2009). Substitutes for procedural fairness: prototypical leaders are endorsed whether they are fair or not. J Appl Psychol.

[111] Steffens NK, Haslam SA, Kerschreiter R (2014). Leaders enhance group members’ work engagement and reduce their burnout by crafting social identity. Ger J Hum Res Manage..

[112] Coakley J (1992). Burnout among adolescent athletes: a personal failure or social problem?. Soc Sport J..

[113] Gustafsson HA, Kenttä GA, Hassmén PA (2011). Athlete burnout: an integrated model and future research directions. Int Rev Sport Exerc Psychol..

[114] Raedeke TD, Lunney K, Venables K (2002). Understanding athlete burnout: coach perspectives. J Sport Behav..

[115] Smith RE (1986). Toward a cognitive-affective model of athletic burnout. J Sport Psychol.

[116] Wegge J, Shemla M, Haslam SA (2014). Leader behavior as a determinant of health at work: specification and evidence of five key pathways. Ger J Hum Res Manage..

